# Prevalence of tooth sensitivity in patients not previously treated periodontally

**DOI:** 10.4317/jced.63219

**Published:** 2025-10-17

**Authors:** María Jesús Martínez-Alcaide, Rocío Marco-Pitarch, Francisco Gil-Loscos, Pedro Almiñana-Pastor, Francisco Alpiste-Illueca, Andrés López-Roldán

**Affiliations:** 1 Department of Stomatology, Faculty of Medicine and Dentistry, University of Valencia, Valencia, Spain; 2 Faculty of Dentistry, Universidad Cardenal Herrera CEU, Valencia, Spain; 3 DD, Post-graduated in Periodontics, Department d´Estomatologia, Facultad de Medicina y Odontologia, Universidad de Valencia, Valencia, Spain; 4 MD DD, PhD in Medicine. Assistant Professor of Periodontics, Department d´Estomatologia, Facultad de Medicina y Odontologia, Universidad de Valencia, Valencia, Spain; 5 Department of Stomatology, School of Medicine and Dentistry, University of Valencia, Valencia, Spain

## Abstract

**Background:**

Dentin hypersensitivity (DH) is a transient pain triggered by thermal, tactile, or osmotic stimuli, commonly linked to exposed dentinal tubules. Its relationship with untreated periodontal disease, however, remains underexplored.

**Material and Methods:**

A retrospective epidemiological study was conducted at a specialized periodontics clinic and included 930 patients diagnosed with periodontal disease. Patients with a history of periodontal treatment, or other potential causes of DH, were excluded. Participants completed a questionnaire assessing habits and symptoms related to DH. Bivariate analyses and statistical tests were used to evaluate the associations between DH and clinical variables such as periodontitis stage, gingival recession, age, gender, toothbrush type, parafunctional habits, and smoking.

**Results:**

DH prevalence was 84.8%, with cold being the most frequently reported stimulus (64.8%). DH increased with the severity of periodontitis, with an odds ratio (OR) of 2.23 for stage IV, grade B periodontitis. Gingival recession was strongly associated with a higher prevalence of DH (88.9% in patients with recession). Women and individuals under 35 reported greater DH. Bruxism and smoking were also contributing factors, particularly in response to sweet or pressure stimuli. The use of soft-bristled toothbrushes increased sensitivity, although not significantly.

**Conclusions:**

DH is highly prevalent in patients with untreated periodontitis and increases with the stage and grade of the disease. Gingival recession, bruxism, and smoking are major contributing factors. Women and younger patients are more susceptible to DH. These findings highlight the need for preventive strategies to manage DH in patients with advanced periodontitis.

## Introduction

Dentin hypersensitivity (DH) refers to a transient sensation or pain in response to thermal, tactile, or osmotic stimuli, which cannot be attributed to any other type of dental pathology (1). Its prevalence ranges from 9% to 52% of the patients (2 - 5), with a higher incidence among individuals undergoing periodontal therapy (2 , 6). The most widely accepted cause of DH is the hydrodynamic theory (7). This theory proposes that external stimuli cause movement of fluid within the dentinal tubules, resulting in changes in intrapulpar pressure that stimulate A-delta nerve fibers, producing pain. For this mechanism to occur, dentin must be exposed and the tubules must remain open and permeable (1). Several factors can lead to the exposure of dentinal tubules, including erosion, abrasion, attrition, abfractions, bleaching agents, certain medications, periodontal surgery, and anatomical factors such as reduced bone or gingival thickness and dental malposition (8 - 10). Gingival recession, in particular, is often associated with DH and is commonly a consequence of periodontal tissue loss. While DH has been extensively studied in patients following periodontal treatment, limited research has focused on its prevalence and associated factors in patients with untreated periodontitis. Understanding this relationship is essential for early diagnosis and prevention strategies, especially in individuals with advanced periodontal disease who have not yet received treatment. Objective: The goal of this study was to evaluate the prevalence of dentin hypersensitivity in patients with untreated periodontitis and to identify associated clinical and behavioral factors.

## Material and Methods

A retrospective epidemiological study was conducted at a specialized periodontics clinic patient records were reviewed for individuals who had undergone a comprehensive periodontal examination, including a periodontal chart and a full-mouth periapical radiographic series. Patients were included if their records contained a confirmed periodontal diagnosis based on the 2018 classification system (11). Exclusion criteria were as follows: History of prior periodontal treatment, active periodontal infections, Stage I-II, Grade A periodontitis, teeth with abscesses, non-vital teeth, fractured teeth, or active caries, presence of any other condition that, in the examiner's judgment, could contribute to dentin hypersensitivity through alternative mechanisms.(All diagnoses were established by an experienced periodontist). Participants completed a structured questionnaire that gathered information on: behavioral factors (e.g., bruxism, smoking), periodontal signs and symptoms (e.g., bleeding, inflammation, gingival recession), oral hygiene practices (e.g., type of toothbrush, bleeding during brushing) and self-reported dentin hypersensitivity (e.g., sensitivity to cold, heat, sweets, and pain triggered by brushing, biting, or pressure) To assess the association between periodontal diagnosis and DH, odds ratio (OR) were calculated using the diagnosis of periodontitis as the exposure factor. Bivariate analyses were conducted to examine associations between DH (general or stimulus-specific) and and clinical/behavioral variables such as gingival recession, toothbrush type, and patient profile characteristics: The Chi-square (²) test was used to evaluate associations between categorical variables. The Mann-Whitney U test was applied to compare two independent samples. For comparisons involving more than two groups, the Kruskal-Wallis test was used. All statistical tests were performed at a significance level of = 0.05. For the ² test (used primarily to evaluate DH prevalence relative to clinical and demographic variables), the statistical power was 0.917, assuming a significance level of 5% and proportions of 0.5 and 0.6 in two homogeneous patient groups.

## Results

The records of a total of 1,353 patient are evaluated. Of these, 423 were excluded due to: incomplete periodontal diagnosis (28), prior periodontal treatment (262), diagnosed with Stage I-II Grade A periodontitis (24), and for meeting one or more of the previously defined exclusion criteria (109). The final study sample comprised 930 patients (n = 930). These 930 patients were classified according to their periodontal diagnosis, (Table 1).

Table 1

**Figure 1:**
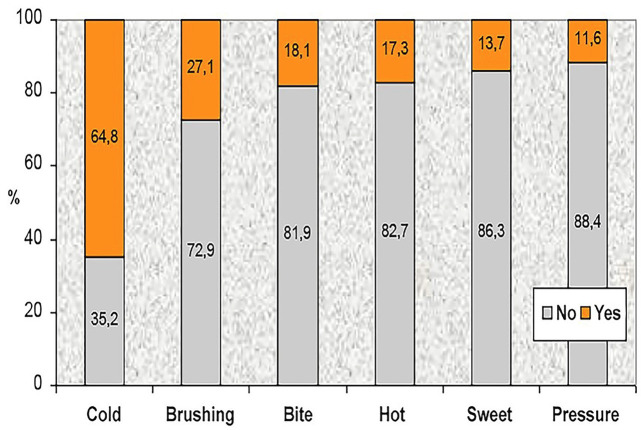
Percentage of DH reported for each stimulus.

The prevalence of DH and the associated triggering stimuli were analyzed in the 930 patients. DH was present in 84.8% of patients diagnosed with periodontitis (809 patients). 'Cold' was the most commonly reported stimulus, affecting 64.8% of the sample. The remaining stimuli presented a considerably lower influence (Fig. 1).

1

When analyzing the number of stimuli experienced simultaneously, 50.3% of patients reported DH triggered by a single stimulus, 30.4% by two stimuli, and 19.4% by three or more. When periodontal diagnosis was combined with the presence of DH, a positive correlation was observed: the prevalence of DH increased with the severity of periodontal disease, namely periodontal stages and grades (p = 0.008, Chi-square test). Table 2 presents the odds ratio (OR) values and corresponding confidence intervals for DH, using Stage I-II Grade B and C periodontitis as the reference diagnosis.

Table 2

**Figure 2:**
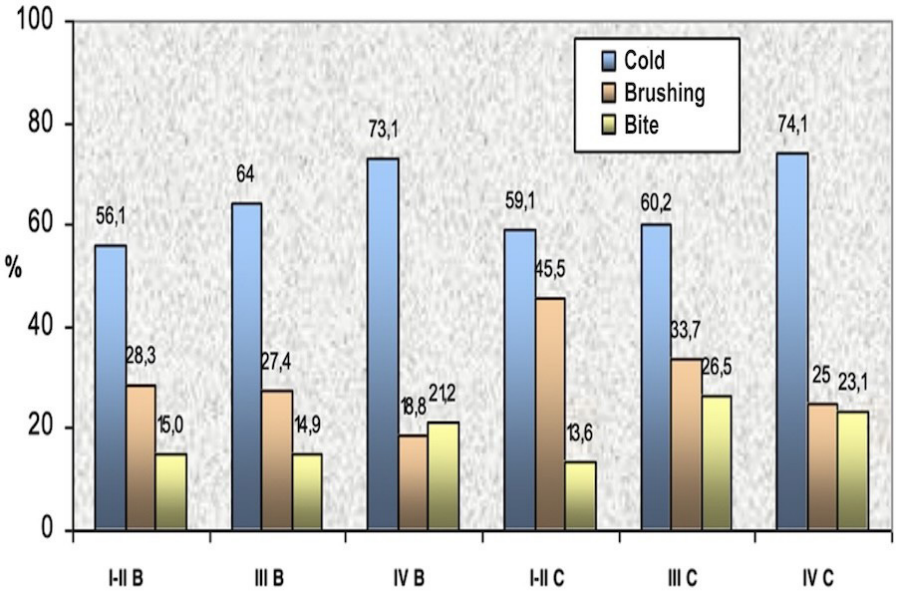
Description of the most significant stimuli according to periodontal diagnosis. (Periodontitis Stages I–II–III–IV and Grades B and C).

The analysis revealed a progressive increase in OR with increasing severity of periodontal disease. Patients with Stage IV Grade B periodontitis had more than twice the odds of experiencing DH compared to those with Stage I-II Grade B (OR = 2.23). Notably, patients with Stage IV Grade C periodontitis exhibited a significantly higher risk, with an OR of 3.63, indicating a 3.6-fold increase in the likelihood of reporting DH. When DH was analyzed in relation to both periodontal diagnosis and the stimulus type, the 'cold' stimulus showed a proportional increase in DH relative to the severity of periodontitis. This association was statistically significant (p = 0.004). In contrast, the 'brushing' stimulus showed an inverse trend: discomfort decreased in patients with more advanced periodontitis (p = 0.021). DH levels in patients diagnosed with Grade C periodontitis were higher than those in Grade B at the same stage. However, the 'biting' stimulus caused a non-significant increase in DH in patients with Grade C (p = 0.056) (Fig. 2).

2

When examining the combined influence of DH etiological factors and triggering stimuli, 'age' emerged as a significant variable for brushing-induced DH (p = 0.003). Patients under 35 years of age reported discomfort more frequently (35.1%) than those aged 36-49 (26.5%) and those over 49 (21%). 'Sex' differences were also notable. Women consistently reported higher rates of DH in response to all stimuli examined. Moreover, they were significantly more likely to experience sensitivity to multiple stimuli simultaneously (p &lt; 0.001) (Table 3).

Table 3

**Figure 3:**
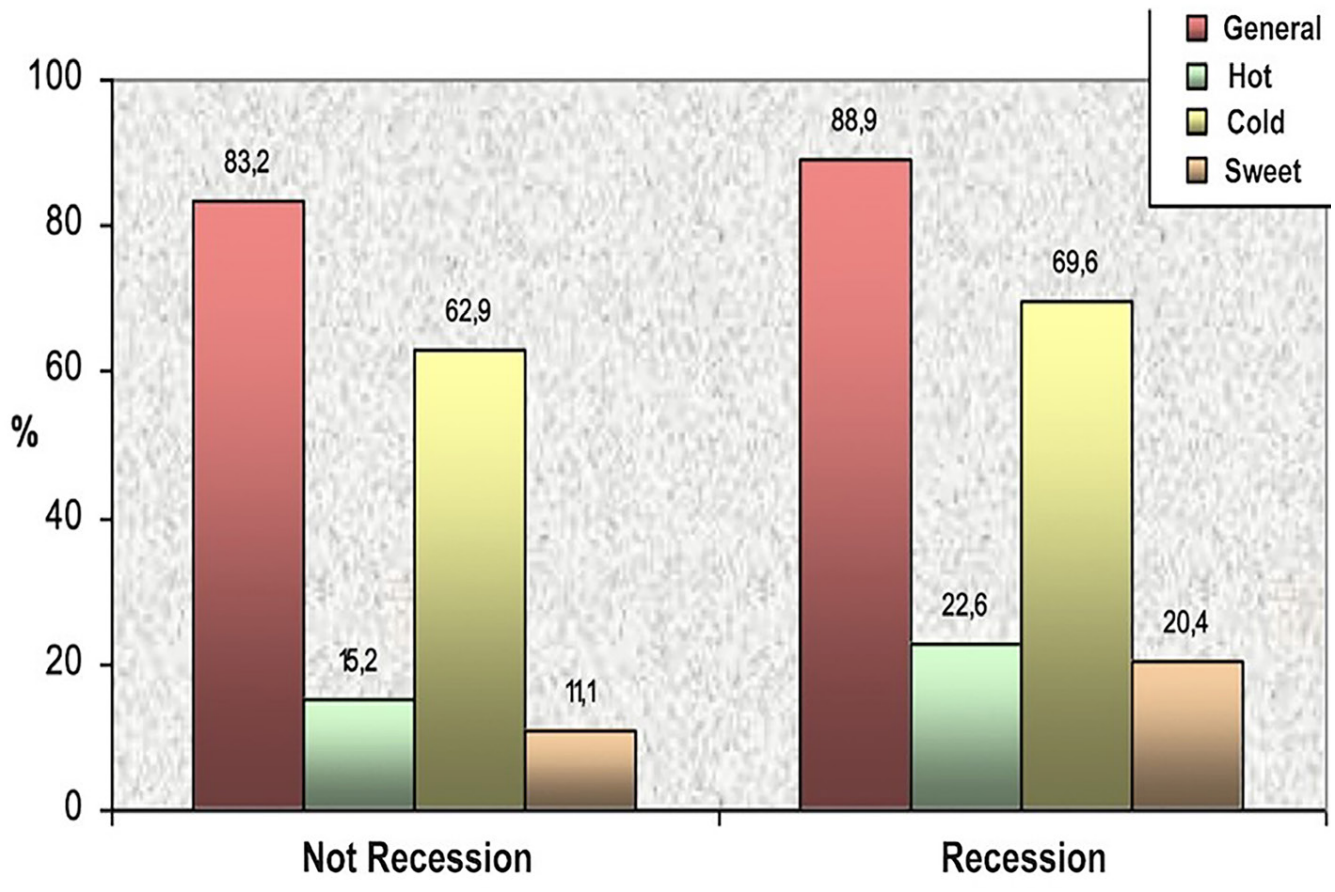
DH prevalence associated to the presence of gingival recession.

'Gingival recession' and DH were strongly correlated. Patients presenting with gingival recession had a significantly higher likelihood of experiencing DH compared to those without recession (p = 0.027), regardless of the stimulus type (Fig. 3).

3

**Figure 4:**
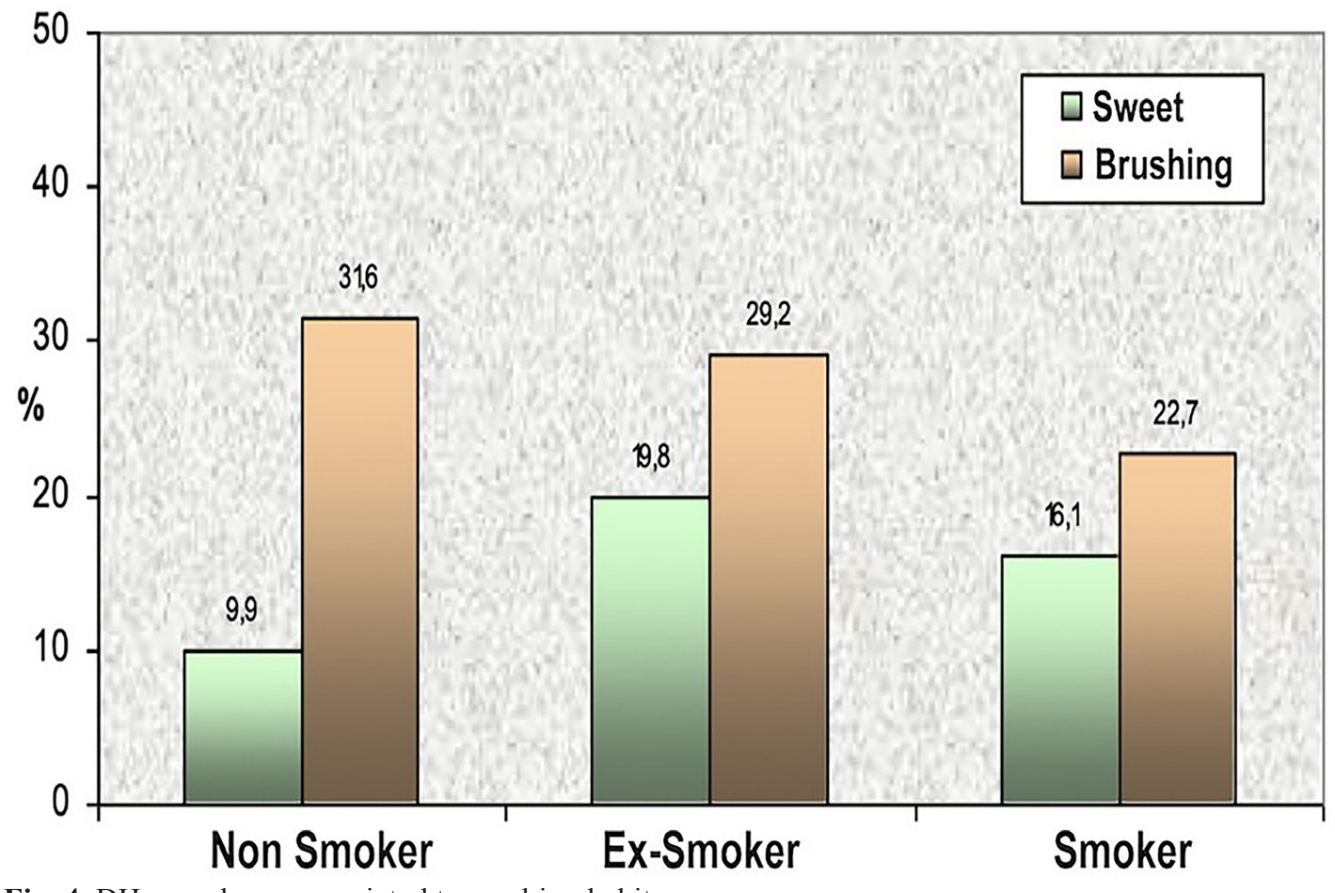
DH prevalence associated to smoking habit.

Among patients with gingival recession, 88.9% reported DH, the prevalence decreased to 83.2% in those without recession. When analyzing the influence of 'toothbrush type', a higher prevalence of DH was observed in users of a soft toothbrush compared to firmer or electric toothbrush; however, this difference was not statistically significant (p= 0.963). 'Bruxism' was also associated with increased DH, particularly in response to sweet stimuli, pressure (p = 0.003), and chewing. In contrast, temperature-related and brushing-induced sensitivity did not differ between patients with and without bruxism. A significant relationship was found between smoking and DH, especially regarding sweet stimuli and brushing. Notably, the number of cigarettes smoked per day influenced brushing-related sensitivity (p = 0.009) (Fig. 4).

4

'Smokers' and 'former smokers' experienced greater DH in response to sweet stimuli, whereas non-smokers reported higher DH levels in response to brushing. Among current smokers, brushing-induced DH decreased with higher cigarette consumption: only 15% of individuals smoking more than one pack per day reported DH in response to brushing, compared to 17.5% (15-20 cigarettes/day), 23.2% (11-15), and 33% (&lt;10 cigarettes/day). No significant association was found between tobacco use and the number of stimuli triggering DH. Multivariate analysis incorporating periodontal diagnosis, DH etiological factors, and stimulus types revealed that gingival recession and bruxism lost statistical significance as independent predictors. However, sex remained a significant factor: women had a 57% higher risk of experiencing DH than men (OR = 1.57). Age was inversely associated with brushing-induced DH, with each additional year of age decreasing the probability by 2.6%. Interestingly, patients with gingival recession had a 38% lower probability of reporting sensitivity to the biting stimulus. Regarding the diagnosis of periodontitis, heat-induced DH was significantly more prevalent in patients with Stage III Grade C and Stage IV Grade C periodontitis, with an odds ratio of 2.07 compared to those with Stage II Grade B. The presence of gingival recession further increased heat sensitivity by 40.1%. For cold stimuli, sensitivity was markedly higher in patients with Stage IV Grade B (88.2%) and Stage IV Grade C (94.8%) periodontitis. Gingival recession amplified this sensitivity by 39.3%. In contrast, no statistically significant differences were observed in DH responses to sweet, biting, or brushing stimuli across different periodontitis diagnoses.

## Discussion

Previous studies on DH have typically focused on its prevalence following periodontal treatment. The present study aimed to analyze the association between DH and untreated periodontitis, examining how disease stage and grade at initial diagnosis influenced sensitivity. Clinical records of 930 patients treated at a specialized periodontal clinic were reviewed. patients with non-periodontal causes of DH, prior periodontal treatment, or exacerbated hypersensitivity were excluded-employing a methodological approach similar to Rees (12). Von Troil et al. conducted a systematic review on the occurrence of DH following periodontal treatment (13), concluding that DH affected approximately half of the patients after basic therapy (scaling and root planing). Symptom intensity typically increased during the initial weeks post-treatment and subsequently declined. Of note, only 23% of patients presented with DH at baseline, a value significantly lower than the 84.8% prevalence observed in the present study. Consistent with our findinds, Chabanski et al. (12) similarly reported a DH prevalence of 84.8% in 507 patients prior to periodontal treatment, based on questionnaire responses. However, their study did not attempt to correlate DH with the periodontal diagnosis. Rees (14) also reported a high DH prevalence (67.7%) following clinical examination and administration of a structured questionnaire. Our study confirms a significant association between DH and periodontitis severity, as defined by both stage and grade, with a statistically significant relationship (p = 0.008). As the severity of periodontal disease increased, so did the prevalence of DH, reinforcing a strong link between disease progression and hypersensitivity. In the present study, a structured questionnaire was conducted alongside the clinical periodontal diagnosis in order to capture the patients' subjective perceptions of DH and the stimuli that triggered it. Previous research has demonstrated that self-reported assessments of DH often yield higher prevalence rates (13-57%) (15 , 16) compared to clinical evaluations (4-32%) (17 , 18), which typically rely on standardized stimuli such as cold air, heat, or mechanical probing. These discrepancies have been attributed to the inclusion of non-specific dental pain in subjective responses, potentially inflating reported prevalence. Our study identified age as a significant factor influencing brushing-induced sensitivity. Younger patients (&lt;35 years) reported the highest levels of DH, with sensitivity decreasing progressively with age. These findings align with those of Dowell (19), who observed greater DH prevalence in younger individuals, a phenomenon attributed to age-related changes in dentin, including increased dentinal sclerosis and reparative dentin formation (20 , 21). Although periodontitis typically progresses with age, DH prevalence remained highest in patients under 35 years (35.1%), compared to those aged 36-49 years (26.5%) and those over 50 (21%). A higher DH prevalence was observed in women, both in general and in terms of sensitivity to multiple stimuli. Rees JS (22) reported a similar trend (143 women vs. 58 men), whereas Al-Wahadni (8) observed higher DH rates in men, attributing this difference to sample variation. Interestingly, although men had a higher prevalence of advanced periodontitis, women reported more frequent and intense DH. This may reflect gender-based differences in oral hygiene habits and healthcare-seeking behavior, as women are more likely to attend dental visits and maintain better oral hygiene (23). Gingival recession showed a strong association with DH in our study: 88.9% of patients with recession reported sensitivity. Recession exposes the root surface, facilitating cementum loss and dentin exposure (24). Al-Wahadni (8) found that 54% of DH patients had gingival recession of 3 mm, compared to only 37% in the control group. This is consistent with Addy's findings (25), which emphasized the multifactorial etiology of DH and the importance of open dentinal tubules for symptom manifestation (26). Patients with Stage III and IV periodontitis presented with more gingival recession than those with milder disease. According to Wennström, Zucchelli, and Pini Prato (27), recession can be classified into three categories: (1) mechanical trauma (e.g., aggressive brushing), (2) localized inflammation, and (3) generalized destruction due to periodontal disease. Our study focused on the third category, as all patients were untreated and had generalized periodontal damage. The loss of interproximal periodontal support in advanced periodontitis leads to compensatory remodelling of buccal and lingual surfaces and apical migration of the gingival margin, thus increasing recession (27). Rees JS and Addy M (28) found a statistically significant relationship (p&lt;0.001) between the high prevalence of gingival recession and periodontal disease, although they did not identify a direct association between recession and the severity of periodontitis. Although our data showed a higher prevalence of DH among users of soft toothbrushes, particularly in response to sweet stimuli, this may reflect reverse causality-patients with existing DH and gingival recession are often advised to switch to soft brushes to minimize trauma (25). Regarding tobacco use, no significant association with DH was observed. While smoking is a well-established risk factor for periodontitis, its role in DH remains unclear. Rees JS (28) reported more sensitive teeth among smokers with periodontal disease, but the difference was not statistically significant. Chabanski (12), using a similar methodology, also found no association between DH and smoking habits or the number of cigarettes consumed. Interestingly, DH to cold and heat stimuli was significantly higher in patients with Stage IV Grade B and C periodontitis, particularly when accompanied by gingival recession. However, no significant associations were observed between type of periodontitis and DH triggered by bruxism, brushing, or tobacco. This study has several limitations. First, the use of self-administered questionnaires introduces subjectivity, as patients may misattribute various forms of dental discomfort to DH. Second, the study sample comprised individuals who actively sought periodontal care, which may limit the generalizability of the findings to the broader population with untreated periodontitis. From a clinical perspective, the findings emphasize the importance of early identification and preventive management of DH in patients with advanced periodontitis (stage III and IV; OR = 3.63), particularly before initiating periodontal therapy. Targeted strategies may help improve patient comfort and quality of life during and after treatment.

## Conclusions

In this study conducted at a specialized periodontics clinic, dentin hypersensitivity (DH) was highly prevalent, affecting 84.8% of the patient population. Cold stimuli were the most commonly reported triggers. The prevalence of DH was positively associated with the severity of periodontal disease, increasing progressively with more advanced stages. Demographic factors also played a significant role: women and younger individuals exhibited the highest rates of DH. Additionally, gingival recession and bruxism were significantly associated with increased DH prevalence, suggesting these factors may contribute to heightened sensitivity in periodontal patients.

## Figures and Tables

**Table 1:** Prevalence percentage of DH across the subgroups of periodontal disease classification (Stages I–II–III–IV and Grades B and C).

		%
TOTAL:	930	100
Periodontitis Stage I-II Grade B	173	18.6%
Periodontitis Stage III Grade B	336	36,1%
Periodontitis Stage IV Grade B	208	22,4%
Periodontitis Stage I-II Grade C	22	2.4%
Periodontitis Stage III Grade C	83	8.9%
Periodontitis Stage IV Grade C	108	11.6%

1

**Table 2:** Prevalence of dentin hypersensitivity (DH) in relation to periodontal diagnosis.

		REFERENCE DIAGNOSIS			
		I-II B		I-II C	
		OR	IC95%	OR	IC95%
Diagnosis	I-II B			0.76	(0.24 2.38)
	III B	1.42	(0.90 2.24)	1.08	(0.53 3.33)
	IV B	2.23	(1.28 3.88)	1.70	(0.53 5.45)
	I-II C	1.31	(0.41 4.09)		
	III C	1.90	(0.92 3.94)	1.45	(0.41 5.10)
	IV C	3.63	(1.62 8.12)	2.77	(0.75 10.2)

2

**Table 3:** Association between DH-inducing stimuli and clinical and sociodemographic parameters relation to periodontal diagnosis.

	Resultsp-value (test)						
		Gender	Gingival recesssion	Type of toothbrush	Bruxismhabit	Smoker	Quantity tobacco
General DH	0,242 (Chi2)	0,181 (Chi2)	0,027 (Chi2)	0,118 (Chi2)	0,677 (Chi2)	0,341 (Chi2)	0,124 (Chi2)
Heat	0,146 (Chi2)	<0,001 (Chi2)	0,007 (Chi2)	0,011 (Chi2)	0,198 (Chi2)	0,134 (Chi2)	0,083 (Chi2)
Cold	0,366 (Chi2)	0,135 (Chi2)	0,049 (Chi2)	0,800 (Chi2)	0,362 (Chi2)	0,132 (Chi2)	0,151 (Chi2)
Sweet	0,681 (Chi2)	0,028 (Chi2)	<0,001 (Chi2)	0,764 (Chi2)	0,003 (Chi2)	0,006 (Chi2)	0,520 (Chi2)
Biting	0,696 (Chi2)	<0,001 (Chi2)	0,571 (Chi2)	0,041 (Chi2)	0,003 (Chi2)	0,493 (Chi2)	0,576 (Chi2)
Brushing	0,003 (Chi2)	0,001 (Chi2)	0,448 (Chi2)	<0,001 (Chi2)	0,061 (Chi2)	0,013 (Chi2)	0,009 (Chi2)
Pressure	0,771 (Chi2)	0,026 (Chi2)	0,089 (Chi2)	0,348 (Chi2)	<0,001 (Chi2)	0,901 (Chi2)	0,050 (Chi2)

3

## Data Availability

The datasets used and/or analyzed during the current study are available from the corresponding author.

## References

[1] Addy M, Urquhart E (1992). Dentine hypersensitivity: its prevalence, aetiology and clinical management. Dent Update.

[2] Chabanski MB, Gillam DG, Bulman JS, Newman HN (1997). Clinical evaluation of cervical dentine sensitivity in a population of patients referred to a specialist periodontology department: a pilot study. J Oral Rehabil.

[3] Demirci M, Karabay F, Berkman M, Tuncer S, Tekçe N (2022). The prevalence, clinical features, and related factors of dentin hypersensitivity in the Turkish population. Clin Oral Investig.

[4] Favaro Zeola L, Soares PV, Cunha-Cruz J (2019). Prevalence of dentin hypersensitivity: Systematic review and meta-analysis. J Dent.

[5] Haneet RK, Vandana LK (2016). Prevalence of dentinal hypersensitivity and study of associated factors: a cross-sectional study based on the general dental population of Davangere, Karnataka, India. Int Dent J.

[6] Taani SDMQ, Awartani F (2002). Clinical evaluation of cervical dentin sensitivity (CDS) in patients attending general dental clinics (GDC) and periodontal specialty clinics (PSC). J Clin Periodontol.

[7] Brannstrom M (1963). A hydrodynamic mechanism in the transmission of pain-producing stimuli through the dentine. Sens Mech Dentine.

[8] Al-Wahadni A, Linden GJ (2002). Dentine hypersensitivity in Jordanian dental attenders. A case control study. J Clin Periodontol.

[9] Liu XX, Tenenbaum HC, Wilder RS, Quock R, Hewlett ER, Ren YF (2020). Pathogenesis, diagnosis and management of dentin hypersensitivity: an evidence-based overview for dental practitioners. BMC Oral Health.

[10] West NX, Lussi A, Seong J, Hellwig E (2013). Dentin hypersensitivity: pain mechanisms and aetiology of exposed cervical dentin. Clin Oral Investig.

[11] Tonetti MS, Greenwell H, Kornman KS (2018). Staging and grading of periodontitis: Framework and proposal of a new classification and case definition. J Periodontol.

[12] Chabanski MB, Gillam DG, Bulman JS, Newman HN (1996). Prevalence of cervical dentine sensitivity in a population of patients referred to a specialist Periodontology Department. J Clin Periodontol.

[13] Von Troil B, Needleman I, Sanz M (2002). A systematic review of the prevalence of root sensitivity following periodontal therapy. J Clin Periodontol.

[14] Rees JS, Jin LJ, Lam S, Kudanowska I, Vowles R (2003). The prevalence of dentine hypersensitivity in a hospital clinic population in Hong Kong. J Dent.

[15] Gillam DG, Seo HS, Bulman JS, Newman HN (1999). Perceptions of dentine hypersensitivity in a general practice population. J Oral Rehabil.

[16] Locker D, Grushka M (1987). The impact of dental and facial pain. J Dent Res.

[17] Taani DQ, Awartani F (2001). Prevalence and distribution of dentin hypersensitivity and plaque in a dental hospital population. Quintessence Int Berl Ger.

[18] Liu HC, Lan WH, Hsieh CC (1998). Prevalence and distribution of cervical dentin hypersensitivity in a population in Taipei, Taiwan. J Endod.

[19] Dowell P, Addy M, Dummer P (1985). Dentine hypersensitivity: aetiology, differential diagnosis and management. Br Dent J.

[20] Mjör IA (1985). Dentin-predentin complex and its permeability: pathology and treatment overview. J Dent Res.

[21] Bissada NF (1994). Symptomatology and clinical features of hypersensitive teeth. Arch Oral Biol.

[22] Rees JS, Addy M (2002). A cross-sectional study of dentine hypersensitivity. J Clin Periodontol.

[23] Addy M (1990). Causas y efectos clinicos de la hipersensibilidad dentinaria. Dent Clin N Am.

[24] Tugnait A, Clerehugh V (2001). Gingival recession-its significance and management. J Dent.

[25] Addy M, Mostafa P, Newcombe RG (1987). Dentine hypersensitivity: the distribution of recession, sensitivity and plaque. J Dent.

[26] Absi EG, Addy M, Adams D (1987). Dentine hypersensitivity. A study of the patency of dentinal tubules in sensitive and non-sensitive cervical dentine. J Clin Periodontol.

[27] Serino G, Wennström JL, Lindhe J, Eneroth L (1994). The prevalence and distribution of gingival recession in subjects with a high standard of oral hygiene. J Clin Periodontol.

[28] Rees JS, Addy M (2004). A cross-sectional study of buccal cervical sensitivity in UK general dental practice and a summary review of prevalence studies. Int J Dent Hyg.

